# A Case of Refractory Variant Angina

**DOI:** 10.7759/cureus.56299

**Published:** 2024-03-16

**Authors:** Yasuhiro Nagayoshi, Miwa Dekita, Masato Nishi, Taiki Nishihara, Kenichi Tsujita

**Affiliations:** 1 Department of Cardiology, Amakusa Medical Center, Amakusa, JPN; 2 Department of Cardiovascular Medicine, Graduate School of Medical Sciences, Kumamoto University, Kumamoto, JPN

**Keywords:** refractory angina treatment, variant angina, pharmacologic spasm provocation test, acute coronary syndrome, coronary artery vasospasm

## Abstract

Coronary vasospasm is defined as the abnormal contraction of an epicardial coronary artery. Variant angina is a severe form of coronary vasospasm, reflecting transmural ischemia with ST-T elevation on an electrocardiogram. A pharmacologic spasm provocation test during coronary angiography is the gold standard evaluation for patients who have not been diagnosed with coronary vasospasm by a non-invasive test. The sensitivity and specificity of pharmacologic spasm provocation testing have been reported to be very high in patients with variant angina. Here, we report the case of a 61-year-old woman who had refractory variant angina. Although a pharmacologic spasm provocation test did not lead to a definitive diagnosis, she had recurrent acute coronary syndrome due to coronary vasospasm. Physicians should be aware of the limitations of the spasm provocation test, even in patients with refractory variant angina.

## Introduction

Coronary vasospasm is defined as the abnormal contraction of an epicardial coronary artery resulting in myocardial ischemia. Variant angina is a severe form of coronary vasospasm, reflecting transmural ischemia with ST-T elevation on an electrocardiogram (ECG). Coronary vasospasm is one of the important etiologies of myocardial infarction with non-obstructive coronary arteries (MINOCAs). The prevalence of MINOCAs is 5%-10% of all myocardial infarctions [1]. Pharmacologic spasm provocation testing during coronary angiography is the gold standard evaluation for patients not diagnosed with coronary vasospasm by a non-invasive test [1, 2]. The sensitivity and specificity of the pharmacologic spasm provocation test have been reported to be very high in patients with variant angina [3]. We present a patient with refractory variant angina who was not diagnosed with the pharmacologic spasm provocation test. 

## Case presentation

**Figure 1:**
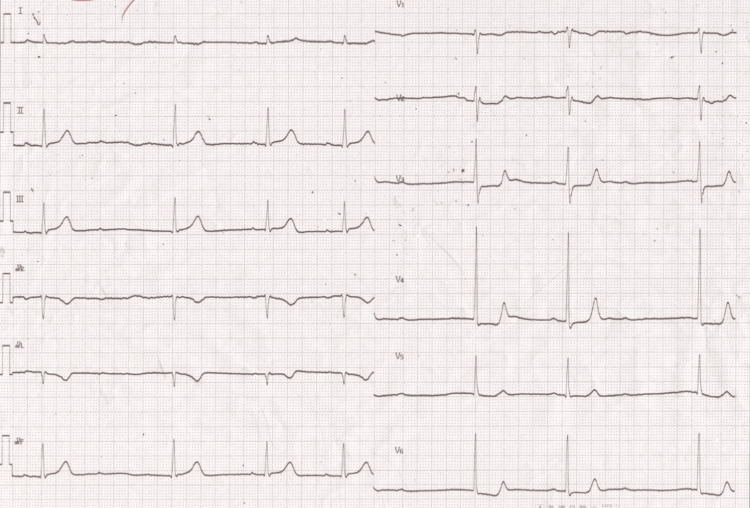
Prehospital twelve-lead electrocardiogram Second-degree atrioventricular block (Wenckebach type) and ST-T elevation in II III aVF leads were observed.

A 61-year-old female sought evaluation at our hospital for early-morning chest discomfort. Her past medical history was unremarkable. The prehospital ECG revealed ST-T elevation in the II, III, and aVF leads (Figure 1). Cardiac troponin I level was 0.39 ng/mL (reference, 0.02-0.06ng/mL). Transthoracic echocardiography revealed severe hypokinesis of the left ventricular posterolateral wall (Video 1).

**Video 1:** Transthoracic echocardiography (short axis) Transthoracic echocardiography revealed severe hypokinesis of the left ventricular posterolateral wall.

**Figure 2:**
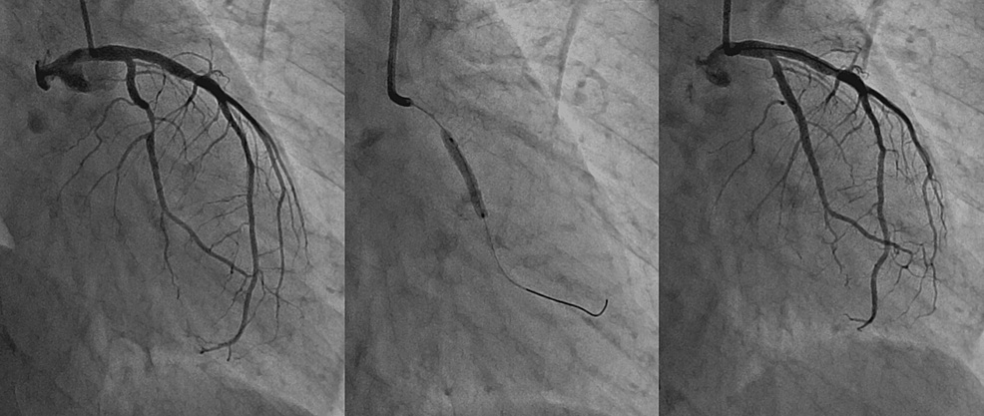
Coronary angiography Coronary angiography showed severe stenosis in the left circumflex artery. Percutaneous coronary intervention with drug-eluting stent implantation (Xience Alpine® 2.75×23mm, Abbott Vascular, Santa Clara, CA, USA) was performed.

After administration of isosorbide nitrate, the ST-T changes and chest discomfort decreased in the catheterization laboratory. Emergent coronary angiography (CAG) showed severe stenosis of the left circumflex artery. The left descending artery and right coronary artery (RCA) were intact. The degree of organic stenosis was severe after intracoronary injection of isosorbide nitrate. We performed percutaneous coronary intervention with a drug-eluting stent ([DES] Xience Alpine® 2.75 × 23 mm; Abbott Vascular, Santa Clara, CA, USA) (Figure 2). The patient had no coronary risk factors, including cigarette smoking. Coronary vasospasm with acute coronary syndrome (ACS) was suspected because the patient had a clinical history of repeated angina at rest between midnight and early morning. Nifedipine (20 mg) was administered to treat the coronary vasospasm in addition to dual antiplatelet therapy and a statin. As the chest pain was not adequately suppressed, diltiazem (100 mg) and isosorbide dinitrate (40 mg) were administered. Left ventricular hypokinesia did not change on follow-up echocardiography.

**Figure 3:**
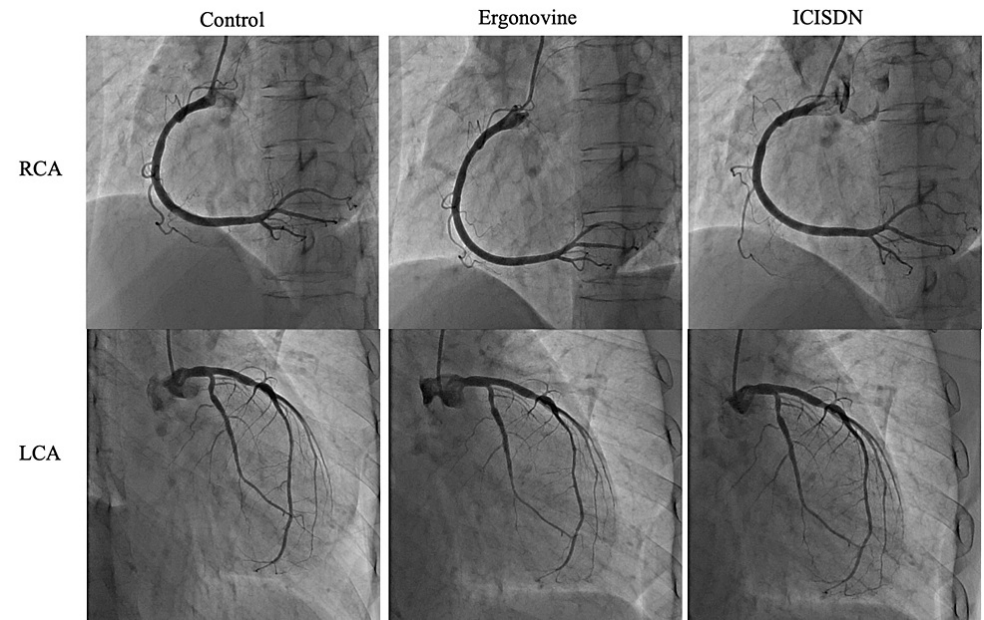
Coronary angiography (Pharmacological spasm provocation test) Coronary vasospasm was not provoked in the right and left coronary artery

**Figure 4:**
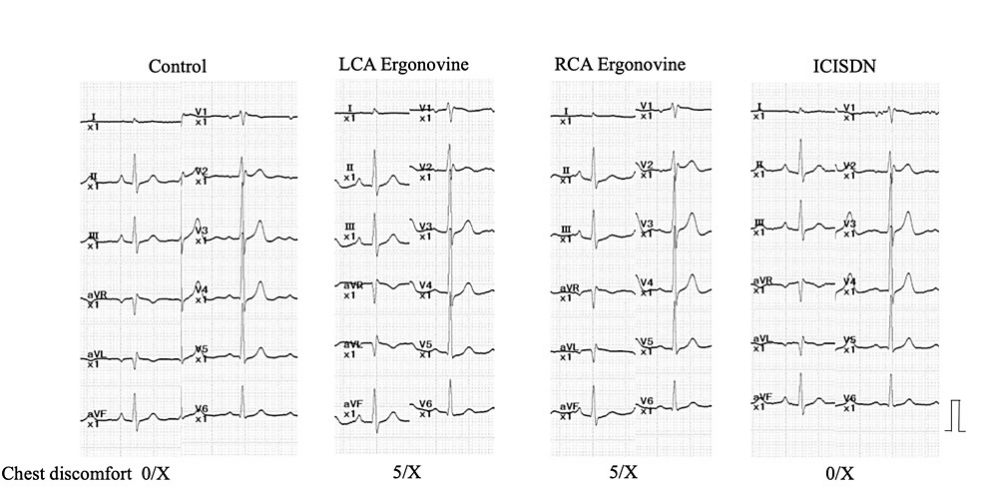
Electrocardiographic changes during pharmacological spasm provocation test ST-T changes were not observed. The patient complained of the same chest discomfort that he had experienced before. ICISDN=intracoronary injection of isosorbide dinitrate LCA= left coronary artery RCA= right coronary artery

Two years later, the patient returned to our hospital due to chest pain. The ECG revealed second-degree atrioventricular block (Wenckebach type) and ST-T elevation in II III aVF leads (ST elevation: III lead > II lead). Sublingual nitroglycerin combined with intravenous diltiazem infusion completely relieved the symptoms and ECG changes. An elective coronary spasm provocation test was planned to confirm the diagnosis of coronary vasospastic angina. Cardiac catheterization was performed 36 hours after discontinuing vasodilator medications, including all calcium blockers and isosorbide dinitrate. The coronary angiogram performed as a control before the spasm provocation test revealed no apparent organic stenosis. Provocation of coronary artery spasm was performed with an intracoronary injection of ergonovine, as previously reported [1]. Ergonovine was injected in incremental doses of 40 μg into the RCA, followed by the injection of 60 μg of ergonovine into the left coronary artery (LCA). Intracoronary injection of ergonovine did not provoke coronary vasospasm with ECG changes in both coronary arteries (Figures 3, 4). The patient complained of chest pain during ergonovine injection and the symptoms resolved after an intracoronary injection of isosorbide dinitrate. As the patient was suspected to have a coronary microvascular spasm, oral nicorandil (15 mg/day) was added to conventional treatments.

**Figure 5:**
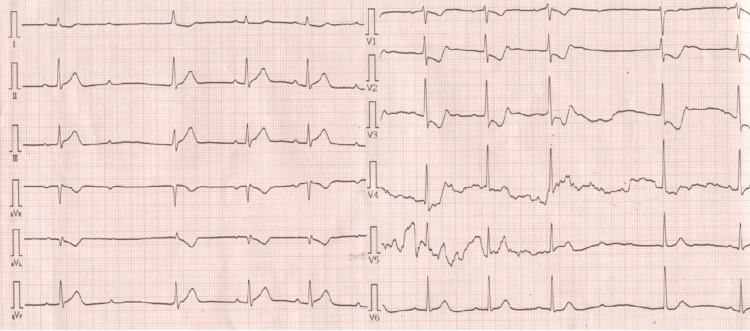
Twelve-lead electrocardiogram in the emergency department Second-degree atrioventricular block (Wenckebach type) and ST-T elevation in II III aVF leads were observed.

The patient sought evaluation at our hospital for a third time with suspected ACS. The ECG revealed second-degree atrioventricular block and ST-T elevation in the II, III, and aVF leads similar to the previous ACS episode (Figure 5). 

**Figure 6:**
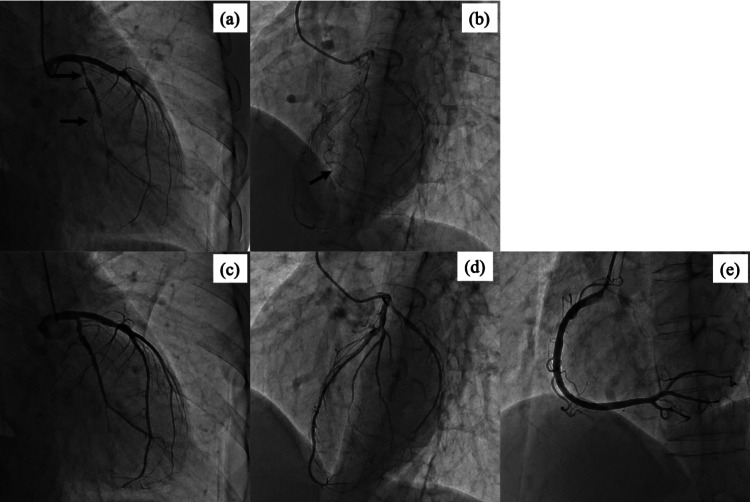
Emergent coronary angiography panel a: Spontaneous coronary vasospasm in the left circumflex (black arrow) panel b: Collateral flow to right coronary artery (black arrow) panel c, d: Left coronary arteriography after intracoronary isosorbide dinitrate infusion Coronary vasospasm and collateral flow to the right coronary artery disappeared. panel e: Right coronary arteriography after intracoronary isosorbide dinitrate infusion

The laboratory findings showed elevation of cardiac enzymes as follows: creatine kinase myocardial band (CK-MB) at 95 IU/L (reference, 0-12 IU/L); and high-sensitive troponin I at 20075.3 pg/mL (reference, 0-46.46 pg/mL). Emergent CAG revealed a spontaneous coronary vasospasm in the left circumflex and collateral flow to the RCA (Figure 6). An intracoronary isosorbide dinitrate infusion resolved the LCA vasospasm and collateral flow to the RCA completely disappeared. Unfortunately, RCA angiography before nitrate administration could not be performed because of bradycardia and hypotension. The patient was diagnosed with refractory variant angina. 

An angiotensin receptor blocker (valsartan at 40 mg/day) was additionally prescribed to prevent left ventricular remodeling after acute myocardial infarction. A detailed medical history revealed that angina attacks tended to occur when the patient's work environment stress increased. Lifestyle modifications to avoid mental stress were recommended. Lifestyle modifications, together with pharmacologic treatments, suppressed recurrent angina attacks.

## Discussion

Refractory vasospastic angina has been typically defined as angina that is not controlled by conventional doses of two or more vasodilators, such as calcium blockers and nitrates [4]. The prevalence of refractory vasospastic angina is approximately 10% among patients with vasospastic angina [5]. Patients with refractory vasospastic angina are generally characterized by a younger age at onset, higher cigarette smoking rates, and normal blood pressure compared to patients with vasospastic angina who respond well to medications [5,6].

The patient described in this report had three ACS episodes. In the first episode, the patient was treated based on elevated troponin I levels and severe hypokinesis of the left ventricular wall. Coronary organic stenosis and coronary artery vasospasm were suspected to be the cause of the ACS episodes. The fractional flow reserve, resting index, and intravascular ultrasound could not be used in the emergent setting. If possible, a spasm provocation test combined with a coronary functional test is ideal to clarify the underlying pathophysiology. This is an important lesson. In the second episode, coronary artery vasospasm could not be proved with an elective spasm provocation test. In the third episode, the ECG changes (ST elevation: III lead > II lead) and the presence of collateral blood flow to the RCA indicated ischemia in the RCA territory, although RCA angiography before administration of isosorbide nitrate could not be performed. LCA angiography revealed a stent-edge spasm in the left circumflex artery. There are several reports about coronary vasospasm associated with drug-eluting stents (DES) [7, 8]. The coronary hyper-constricting response is enhanced at the DES edge. The potential underlying mechanisms include DES-related endothelial dysfunction and Rho-kinase activation, which is a molecular switch that regulates vascular smooth muscle contraction. 

There are several possible reasons why the pharmacologic spasm provocation test failed to demonstrate coronary vasospasm. Firstly, the period of vasodilator discontinuation might be insufficient in our patient. According to the Japanese Circulation Society guidelines, a vasodilator washout period of 2 days or longer is recommended to increase the diagnostic accuracy of the drug-induced spasm provocation test [1,5]. Sueda et al. reported the clinical usefulness of performing the spasm provocation test under medical therapy in patients with refractory coronary vasospastic angina [9]. It was unlikely that the period of vasodilator withdrawal was the only reason coronary vasospasm was not demonstrated. Secondly, low-disease activity can lead to false-negative results but the patient had refractory variant angina with high-disease activity. Finally, the characteristics of the spasm-provocative drugs might have contributed to the findings. The pharmacologic spasm provocation test is performed with acetylcholine or ergonovine. Ergonovine acts on vascular smooth muscle via serotonergic receptors and alpha-adrenergic receptors, while acetylcholine acts via muscarinic cholinergic receptors. Although the sensitivity of these mediators is thought to be comparable, acetylcholine is reported to be more sensitive in females [10]. Different mediators and doses might induce coronary vasospasm. The spasm provocation test, when using a combination of ergonovine with acetylcholine, is a method to increase test sensitivity [11]. Irrespective of the method used, however, physicians should keep in mind that there are limits to the pharmacologic provocation of coronary vasospasm.

Refractory angina attacks were stabilized with multiple vasodilators and lifestyle modifications. Mental stress was strongly associated with angina attacks in our patient. Rho-kinase activity in circulating neutrophils has been reported to be enhanced in states of high mental stress [12]. Avoidance of mental stress might have a central role in angina attack suppression. In addition, the administration of an ARB might contribute in part to a reduction of angina attacks by another mechanism. Oxidative stress impairs vascular endothelial function, resulting in decreased nitric oxide production and precipitation of coronary vasospasm. Angiotensin II induces the generation of reactive oxygen species via NADPH oxidase activation. Renin-angiotensin inhibitor therapy is associated with a lower incidence of cardiovascular events, including recurrent angina that requires follow-up CAG in patients with coronary spastic angina [13,14]. This theory remains a matter of speculation in our patient because oxidative stress markers were not measured.

Polymorphisms in endothelial NOS, aldehyde dehydrogenase 2, and paraoxygenase-1 genes have been reported to be associated with coronary vasospasm [15]. The -786T/C polymorphism in the 5’-flanking region of the endothelial nitric oxide synthase gene allele is an independent risk factor for readmission due to a recurrent vasospastic angina attack [16]. These candidate genetic disorders could not be examined in our patient.

## Conclusions

The spasm provocation test is not a perfect method for a definitive diagnosis of coronary vasospasm, even in patients with refractory variant angina. Physicians should be aware of the limitations of the pharmacologic spasm provocation test.
